# The prognostic value of combining MNA-SF nutritional status and probable sarcopenia for 90-day readmission in elderly inpatients with multimorbidity

**DOI:** 10.3389/fmed.2026.1862824

**Published:** 2026-09-09

**Authors:** Jinhua Sun, Fenglin Li, Yuanyuan Sun

**Affiliations:** Department of Clinical Nutrition, The Second People’s Hospital of Hefei (Hefei Hospital Affiliated to Anhui Medical University), Hefei, China

**Keywords:** aged, malnutrition, multimorbidity, probable sarcopenia, prognosis, readmission

## Abstract

**Objective:**

To explore the predictive value of nutritional status combined with Probable sarcopenia assessment for all-cause readmission of elderly inpatients with multimorbidity within 90 days after discharge.

**Methods:**

In this single-centre retrospective cohort study, a total of 244 patients aged ≥65 years admitted to our hospital from January 2023 to March 2025 were selected as the research subjects. Abnormal nutritional status (malnutrition or risk of malnutrition, MNA-SF ≤11) and probable sarcopenia (per modified EWGSOP2-based criteria) were assessed within 48 h of admission, and patients were divided into a readmission group (59 cases) and a non-readmission group (185 cases) according to whether they were readmitted within 90 days after discharge.

**Results:**

The age of patients in the readmission group was significantly higher than that in the non-readmission group (*P* < 0.05), the Charlson Comorbidity Index was higher (*P* < 0.05), the level of C-reactive protein was higher (*P* < 0.05), and the serum albumin, handgrip strength and calf circumference were lower (*P* < 0.05). The overall prevalence of probable sarcopenia was 31.1%, and the prevalence of malnutrition (including risk) was 73.8%. Abnormal nutritional status was defined as MNA-SF ≤11 (combining at-risk and malnourished categories). The combined group analysis showed that the 90-day readmission rate of patients with abnormal nutritional status and probable sarcopenia (Group D) was the highest, reaching 47.8%, which was significantly higher than that of other groups (*P* < 0.05). Multivariate logistic regression analysis showed that after adjusting for age, sex, comorbidities and inflammatory markers, the combined status was independently associated with higher odds of 90-day readmission (aOR=3.94, 95%CI: 1.73–8.96, *P* = 0.001). The combined assessment model demonstrated moderate discriminative ability (AUC 0.724, 95% CI: 0.651–0.797), which was statistically significantly better than that of nutrition or probable sarcopenia assessment alone (DeLong *P* < 0.05).

**Conclusion:**

In this single-center retrospective cohort, the coexistence of abnormal nutritional status (MNA-SF ≤11) and probable sarcopenia was independently associated with higher odds of 90-day readmission in elderly hospitalized patients with multimorbidity. Larger prospective multicenter studies are needed to validate the combined assessment and determine whether targeted interventions reduce readmission.

## 1 Introduction

Readmission of elderly patients with multimorbidity is a growing challenge for healthcare systems worldwide. Unplanned rehospitalisation within 30–90 days occurs in approximately 15%–25% of older medical inpatients and is associated with functional decline, increased caregiver burden, and higher healthcare costs. Established risk factors include advanced age, high comorbidity burden, polypharmacy, functional dependence, and social vulnerability. Among potentially modifiable patient-level factors, nutritional deficits and muscle wasting have received increasing attention because of their high prevalence and strong link with adverse post-discharge outcomes (1). Among many influencing factors, malnutrition and sarcopenia have attracted much attention due to their high prevalence in elderly inpatients and strong correlation with poor prognosis (2). As an effective screening tool, the Mini-Nutritional Assessment-Short Form (MNA-SF) has been widely used to identify patients at risk of malnutrition or in a state of malnutrition (3). Research shows that malnutrition at the time of hospitalization is an important predictor of short-term and long-term mortality, complications and increased utilization of healthcare resources (4). At the same time, sarcopenia, a syndrome characterized by progressive and extensive decline in skeletal muscle mass, strength and function, has also been proved to be an independent risk factor for the loss of independence, increased fall risk and increased mortality in elderly patients (5). The diagnostic pathway recommended by the European Working Group on sarcopenia in Older People 2 (EWGSOP2) in 2019 provides a practical framework for clinical practice from screening to diagnosis (6). It is worth noting that malnutrition and sarcopenia often co-exist. They share pathophysiological bases such as inflammation, metabolic changes and anorexia, forming a vicious cycle of “malnutrition-inflammation-sarcopenia” (7). Existing evidence shows that in internal medicine inpatients, the existence of these two conditions at the same time is significantly correlated with worse in-hospital outcomes (8). In addition, in the specific group of patients with heart failure, malnutrition is often associated with more significant inflammatory reaction and fluid retention (congestion), and these factors themselves are the driving factors of poor prognosis (9). These findings suggest that evaluating nutrition and muscle status may provide key information for predicting the clinical trajectory of elderly vulnerable patients, especially the risk of readmission.

Although existing studies have established malnutrition and sarcopenia as independent predictors of poor prognosis, there are still some limitations (10). First of all, most studies tend to analyze the impact of nutritional status or muscle function on outcomes independently, and fail to fully quantify the synergistic or additive effects of the two coexistence (11). Secondly, in terms of assessment methods, there are differences in nutritional screening tools and diagnostic criteria for sarcopenia used in different studies, which to some extent affects the homogeneity and comparability of research results (12). Moreover, the prognostic value of the combined status of the two, especially after adjusting for key confounding factors such as comorbidity burden and systemic inflammation level, whether it is an independent risk factor for readmission, still needs to be further verified in larger, prospective multicentre studies (13). Finally, in routine clinical practice, the systematic simultaneous nutritional screening and Probable sarcopenia assessment for hospitalized elderly patients has not been popularized, partly due to the lack of concise, efficient and prognostically validated combined assessment strategies (14). Therefore, to build a combined risk stratification model that combines the validated nutritional screening tool (such as MNA-SF) and the practical sarcopenia assessment pathway (such as EWGSOP2 pathway), and to clarify its predictive efficiency for short-term readmission, an important public health indicator, has clear clinical needs and scientific research value (15).

In view of this, this study specifically investigates whether a straightforward combined phenotype—abnormal nutritional status (MNA-SF ≤11) plus EWGSOP2-based Probable sarcopenia—enhances 90-day readmission risk stratification of older multimorbid medical inpatients beyond either domain alone, after adjusting for key confounders. The contribution of this study lies in applying this combined phenotype to a single-center cohort of elderly multimorbid medical inpatients with a 90-day readmission outcome, using assessment tools readily available in clinical practice to construct a four-category combined exposure variable and thereby distinguish the influence of a single factor from the coexistence state. We hypothesized that the coexistence of malnutrition and Probable sarcopenia would significantly increase the risk of short-term readmission, and this association was independent of traditional risk factors such as age, comorbidity and inflammation. By clarifying the prognostic significance of this combined phenotype, this study is expected to provide evidence-based evidence for early identification of high-risk patients and implementation of targeted interventions.

It is acknowledged that 90-day readmission is a complex endpoint influenced not only by biological vulnerability but also by clinical severity at discharge, quality of discharge planning, social support, access to ambulatory care, and healthcare policy. Nevertheless, nutritional status and muscle function represent potentially modifiable patient-level factors that may affect post-discharge recovery capacity, susceptibility to complications, and functional decline—all of which can precipitate rehospitalization independent of healthcare system factors. Therefore, identifying patients with combined nutritional and sarcopenic impairment at admission may capture a dimension of vulnerability that complements clinical severity measures and informs targeted discharge planning.

## 2 Data and methods

### 2.1 General information and sample size

This study was a single-centre, retrospective cohort study. We retrospectively collected the baseline data of elderly inpatients who were continuously admitted to the internal medicine ward of our hospital from January 1, 2023 to March 31, 2025, and confirmed the readmission outcomes 90 days after discharge. The selection of study time window aims to include a sufficient number of cases and ensure sufficient follow-up time to observe the primary outcome. The sample size was estimated based on the expected effect size. Sample size was estimated for the primary logistic regression analysis. Based on previous literature, we assumed: (1) a 90-day readmission event rate of approximately 24%; (2) an exposure prevalence of 25% for the combined malnutrition-Probable sarcopenia group (Group D); and (3) an odds ratio of at least 3.0 for Group D vs. Group A. With *α* = 0.05 (two-sided) and power (1-β) = 0.80, a minimum of 220 patients was required (PASS 15.0 software). The final sample of 244 patients met this requirement. A total of 259 patients met all inclusion and exclusion criteria and constituted the initial study cohort. During the index hospitalization, 15 patients (5.8%) died. These patients were not at risk for post-discharge readmission and were therefore excluded from the primary 90-day readmission analysis. Consequently, the final analytic sample for the primary outcome comprised 244 patients who were discharged alive. For the secondary composite outcome of death or readmission within 90 days, the entire initial cohort of 259 patients was analyzed, with in-hospital deaths counted as events. Initially, 312 elderly inpatients admitted to the internal medicine ward were screened.

**Figure 1:**
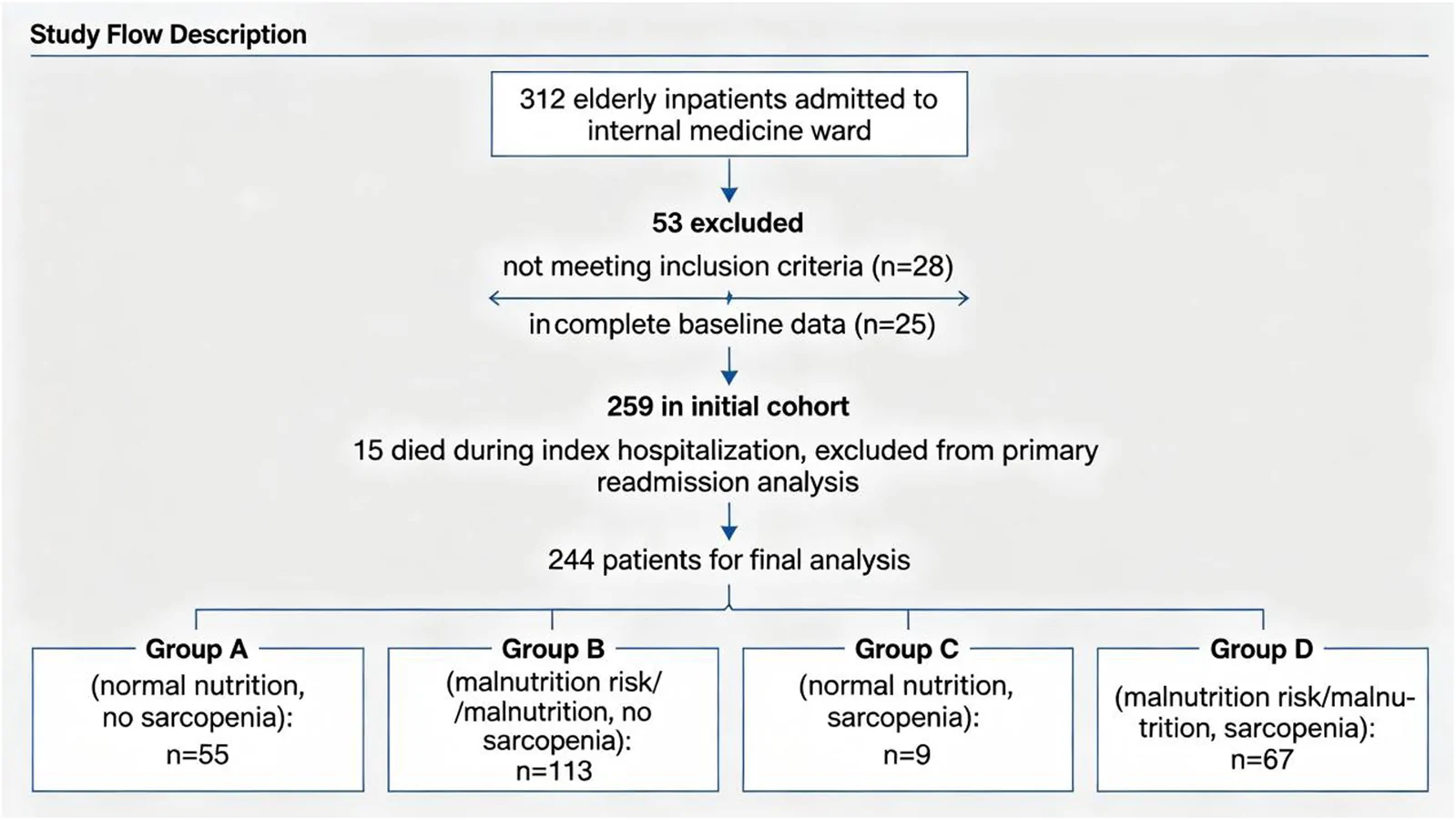
Research roadmap.

53 were excluded: not meeting inclusion criteria (*n* = 28) or incomplete baseline data (*n* = 25), leaving 259 in the initial cohort. During index hospitalization, 15 died and were excluded from the primary readmission analysis, yielding 244 patients for final analysis. See Figure 1.

### 2.2 Inclusion and exclusion criteria

In order to ensure the homogeneity of the study population and the interpretability of the results, we developed clear inclusion criteria.

1. Inclusion criteria: ① age ≥65 years old; ② Admitted to the general internal medicine ward of our hospital due to acute medical diseases (such as acute heart failure, pneumonia, acute exacerbation of chronic obstructive pulmonary disease, etc.); ③ The length of stay was more than 48 h to ensure that there was sufficient time to complete the systematic assessment at the initial stage of admission; ④ Within 48 h after admission, the Mini-Nutritional Assessment-Short Form (MNA-SF) scores and Probable sarcopenia-related assessments (such as handgrip strength, calf circumference or SARC-F questionnaire records) were completely recorded in the medical records.
  For the purpose of this study, multimorbidity was defined as the presence of two or more chronic conditions that were recorded in the medical history and corresponded to the disease categories of the Charlson Comorbidity Index. This definition has been widely used in geriatric research and reflects the coexistence of multiple chronic diseases.
2. Exclusion criteria: ① patients in the state of hospice care or with a life expectancy of less than 3 months as judged by the attending physician; ② Those who suffer from severe cognitive impairment (such as moderate or severe dementia), mental illness or disturbance of consciousness, resulting in failure to cooperate reliably in completing nutritional or functional assessment; ③ The key data in the electronic medical record (such as MNA-SF score, handgrip strength value, discharge date, primary diagnosis, etc.) are missing more than 20%, which cannot be effectively analyzed; ④ Patients who are lost to follow-up after discharge and cannot confirm their readmission status within 90 days through the hospital information system or telephone follow-up.

### 2.3 Equipment and materials

All the equipment and materials used in this study are routine clinical applications to ensure the authenticity and repeatability of the data.

1. Winning Health Clinical Information System 5.6: as the core data source, it is used to extract all demographic information, diagnosis, laboratory test results, nursing evaluation records and readmission information of patients.
2. Handgrip strength meter: Camry EH101 electronic handgrip strength meter (Guangdong Xiangshan Weighing Instrument Group Co., Ltd.) is used to measure handgrip strength. The measuring range of the equipment is 0–90 kg and the accuracy is 0.1 kg, which has passed the annual metrological calibration. During the measurement, the patient took the sitting position, elbow flexion 90°, forearm neutral position, and continuously measured the dominant hand grip strength three times. The maximum value was recorded in kilograms (kg). This method is an international standard for evaluating muscle strength.
3. Soft tape measure: use medical grade inelastic soft tape measure (length 150 cm, minimum scale 1 mm) to measure the calf circumference. The patient was in the supine position, and his legs were naturally extended. The circumference of the thickest part of the non-dominant leg was measured to the nearest 0.1 cm (cm). Calf circumference is a simple and effective index for screening muscle mass in community and clinical settings.
4. Standardized assessment tools: including Mini-Nutritional Assessment-Short Form (MNA-SF), SARC-F questionnaire, Barthel Index (BI) assessment form, etc. These are internationally recognized scales with Chinese validated versions, which are integrated into the admission assessment process of our hospital in the form of paper or electronic forms.

### 2.4 Research methods

1. Study design framework: This was a retrospective cohort study that utilized baseline records collected during the index hospitalization and subsequent ascertainment of 90-day readmission outcomes from the hospital information system and telephone follow-up. The temporal sequence between exposure assessment (at admission) and outcome (post-discharge) was preserved, supporting the identification of independent associations.
2. Data collection and quality control: Two research nurses who were familiar with internal medicine diseases and blinded to patient outcomes independently extracted data from electronic medical records using a pre-designed standardized data extraction form. The extracted content covered all observation indicators in Section 2.5. For continuous variables (such as handgrip strength and laboratory indicators), record the values directly; for the scale scores, check the original evaluation records. After data extraction, cross-checking was performed, and inconsistencies (incidence rate <5%) were adjudicated by a third senior research physician (with 10 years of clinical experience in internal medicine) with reference to the original medical records. The final data were entered into the EpiData 4.6 database in the form of double entry and logical verification.
3. Definition and grouping of core exposure variables: Nutritional status assessment: strictly follow the official scoring standard of the Mini-Nutritional Assessment-Short Form (MNA-SF). The scale includes 6 items: weight loss, eating situation, activity ability, psychological stress, neuropsychiatric problems and body mass index (BMI) or calf circumference. The total score is 14 points. According to the consensus, we divided patients into three groups: normal nutrition group (12–14 points), at risk of malnutrition group (8–11 points), and malnourished group (0–7 points). This classification has been confirmed by many studies to be effective in predicting adverse clinical outcomes.

Diagnosis of probable sarcopenia: Because dual-energy x-ray absorptiometry (DXA) or bioelectrical impedance analysis (BIA) was not available in our clinical routine, direct quantification of muscle mass could not be performed. Therefore, participants could not be classified as having confirmed sarcopenia according to the EWGSOP2 algorithm. Instead, we operationally defined probable sarcopenia by the simultaneous presence of (i) a positive SARC-F screening (score ≥4), (ii) low handgrip strength (<28 kg for men, <18 kg for women, based on AWGS 2019 (16) cut-offs), and (iii) low calf circumference (<34 cm for men, <33 cm for women). This conservative three-criterion approach was chosen to increase specificity in the absence of direct muscle mass measurement, although it likely reduces sensitivity for detecting true probable sarcopenia as defined by EWGSOP2 (low muscle strength alone). We recognise that this is a substantial deviation from EWGSOP2 and that the diagnostic performance of this specific combination has not been formally validated against a reference standard; therefore, throughout the manuscript we refer to this phenotype as ‘probable sarcopenia’ rather than confirmed sarcopenia.

Combined exposure grouping: To explore the combined effect of nutritional status and Probable sarcopenia, we created a four-category variable: Group A (reference group): normal nutrition (MNA-SF ≥12) and no Probable sarcopenia; Group B: with malnutrition risk or malnutrition (MNA-SF ≤11), but without Probable sarcopenia; Group C: normal nutrition, but with Probable sarcopenia; Group D: with malnutrition risk/malnutrition (MNA-SF ≤11) and Probable sarcopenia simultaneously. The multiplicative interaction between abnormal nutritional status and probable sarcopenia was not statistically significant (*P* = 0.312). The higher readmission rate in Group D thus reflects the joint association of two frequently co-existing conditions rather than a synergistic biological interaction. We therefore describe this as a joint association.

For the primary analysis, MNA-SF was scored using BMI as the anthropometric component whenever available; calf circumference was used as a substitute only when BMI could not be measured (*n* = 18 patients). To address potential measurement overlap between nutritional and Probable sarcopenia assessments, we conducted a sensitivity analysis using MNA-SF scored without the calf circumference/BMI item (recalibrated to a maximum of 11 points), with abnormal nutrition defined as score ≤8.

Calf circumference measurement was standardized to minimize bias from fluid status: measurements were performed in the morning, with the patient supine for at least 30 min. The presence of bilateral pitting edema was recorded. For patients with bilateral grade ≥2 pitting edema (*n* = 34), calf circumference measurements were interpreted with caution, as fluid retention may overestimate true muscle mass. In a *post-hoc* sensitivity analysis excluding patients with significant edema, the prevalence of Probable sarcopenia was 28.7% vs. 31.1% in the full cohort, suggesting a modest potential for misclassification.

4. Follow-up and outcome determination: The primary outcome was readmission for any reason within 90 days after discharge (to our hospital or other hospitals with clear referral records). The outcome information was confirmed in two ways: first, the readmission records of all patients in the hospital information system (HIS) of our hospital within 90 days after discharge were queried; secondly, for patients with no readmission record in HIS, trained research staff conducted telephone follow-up 90–100 days after discharge. A standardised script was used to inquire about any hospitalisation at other institutions. At least three attempts were made on different days and at different times. If the patient could not be reached, a designated family caregiver (recorded at admission) was contacted. The outcome was verified by cross-checking with any available referral records or discharge summaries from the outside hospital, if provided by the family. Through this protocol, follow-up was successfully completed for all 244 patients, yielding a 100% response rate for the primary outcome. While this rate is high, we note that the cohort consisted of patients from a relatively stable urban catchment area with high telephone accessibility, which may have contributed to the completeness of follow-up. Readmission status was independently determined by two researchers, and differences were resolved through discussion.

### 2.5 Observational indicators

We collected the following observational indicators to comprehensively describe patient characteristics, define exposure, and control confounding:

1. Primary outcome measure: 90-day all-cause readmission (yes/no), assessed in the 244 patients discharged alive.
2. Secondary outcome measures: ① All-cause in-hospital mortality (*n* = 15/259, 5.8%); ② Total length of stay for the index hospitalization; ③ Composite endpoint of death or readmission within 90 days of the index admission date, assessed in the full initial cohort of 259 patients. For this composite endpoint, death during the index hospitalization or within 90 days of discharge counted as an event.
3. Core exposure indicators: ① nutritional risk screening score (MNA-SF, continuous variable and categorical variable); ② sarcopenia screening score (total score of SARC-F questionnaire); ③ absolute handgrip strength (kg); ④ calf circumference (cm).
4. Key covariate indicators (for multivariate model adjustment):
  4.1 Demographic basis: age (years) and sex.
  4.2 Disease burden and severity: Charlson Comorbidity Index (CCI), calculated according to admission diagnosis; primary admission diagnosis categories (heart failure, infection, respiratory diseases, etc.); the National Early Warning Score 2 (NEWS2) at admission was used to quantify the severity of acute diseases.
  4.3 Biochemical markers of inflammation and nutrition: serum albumin (ALB, g/L) and C-reactive protein (CRP, mg/L) detected for the first time within 72 h after admission. We also calculated the albumin to globulin ratio (A/G) as a comprehensive indicator of nutritional and inflammatory status. Previous studies have suggested that it is related to prognosis (1).
  4.4 Functional status and body measurement: Barthel Index (BI) assessed the ability of basic activities of daily living, with a total score of 100. The lower the score, the stronger the dependence; body mass index (BMI, kg/m²).
  4.5 Other clinical related indicators: polypharmacy, defined as daily long-term use of ≥5 prescription drugs; whether there was dysphagia at admission (evaluated and recorded by the nurse); whether there was pressure injury (according to nursing records).

### 2.6 Statistical methods

All statistical analyses were performed using IBM SPSS Statistics version 26.0 (IBM Corp., Armonk, NY, USA) software. First, the patients were divided into a “readmission group” and a “non-readmission group” according to whether they were readmitted within 90 days. The normality of measurement data was evaluated by the Shapiro–Wilk test. Variables conforming to normal distribution were described by mean ± SD, and the independent sample t-test was used for comparison between groups; non-normally distributed variables were described by median (interquartile range) [M (IQR)], and the Mann–Whitney U test was used for comparison between groups. Categorical data were expressed as number of cases (percentage) [n (%)]. The chi-square test or Fisher’s exact test was used for comparison between groups (when the expected frequency was <5).

To evaluate the independent predictive value of combined nutritional status-Probable sarcopenia grouping for 90-day readmission, we constructed a multivariate binary logistic regression model. The dependent variable was “readmission within 90 days”. The core independent variable was the four-category combined exposure group (with Group A (normal nutrition without Probable sarcopenia) as the reference). The final adjusted covariates included age (continuous variable), sex, Charlson Comorbidity Index (continuous variable), log-transformed admission CRP value (to deal with its skewed distribution), and admission albumin value (continuous variable). The National Early Warning Score 2 (NEWS2) was not included in the primary model because it is an acute severity-of-illness score measured at a single time point and was strongly correlated with both CRP (Spearman’s *ρ* = 0.52, *P* < 0.001) and the combined exposure groups (*P* < 0.001), raising concerns about collinearity and overadjustment. In a pre-planned sensitivity analysis, NEWS2 was added to the fully adjusted model to evaluate the stability of the effect estimates. We quantified the association strength by calculating the adjusted odds ratio (aOR) and its 95% confidence interval (95% CI). In addition, in order to preliminarily explore whether there is a statistical interaction between nutritional status (binary classification: MNA-SF ≤11 vs. >11) and Probable sarcopenia (binary classification: yes vs. no), we introduced the product term of the two into the logistic regression model containing the main effects, and conducted the likelihood ratio test. Given the 59 readmission events and 9 estimated parameters (approximately 6.6 events per variable), we acknowledge the potential for overfitting. To assess model optimism, we performed bootstrap internal validation with 1000 resamples. Specifically, we drew 1000 bootstrap samples of size n = 244 with replacement from the original dataset. For each bootstrap sample, the full logistic regression model was fitted, and its predictive performance was evaluated both on the bootstrap sample (apparent performance) and on the original dataset (test performance). Optimism was calculated as the difference between the bootstrap apparent performance and the test performance for each resample, and the mean optimism across all 1000 resamples was subtracted from the apparent AUC of the original model to obtain the optimism-corrected AUC. The optimism-corrected calibration slope was calculated analogously. Model calibration was assessed by the Hosmer–Lemeshow goodness-of-fit test, and a calibration plot was generated by plotting the observed readmission probabilities against the predicted probabilities from the combined model, with a locally weighted scatterplot smoothing (LOWESS) curve overlaid to visually evaluate agreement. The optimism-corrected AUC and calibration slope are reported alongside the apparent model performance. All hypothesis tests were two-sided, and *P* < 0.05 was considered statistically significant.

## 3 Results

### 3.1 Comparison of baseline characteristics between readmission group and non-readmission group

**Figure 2:**
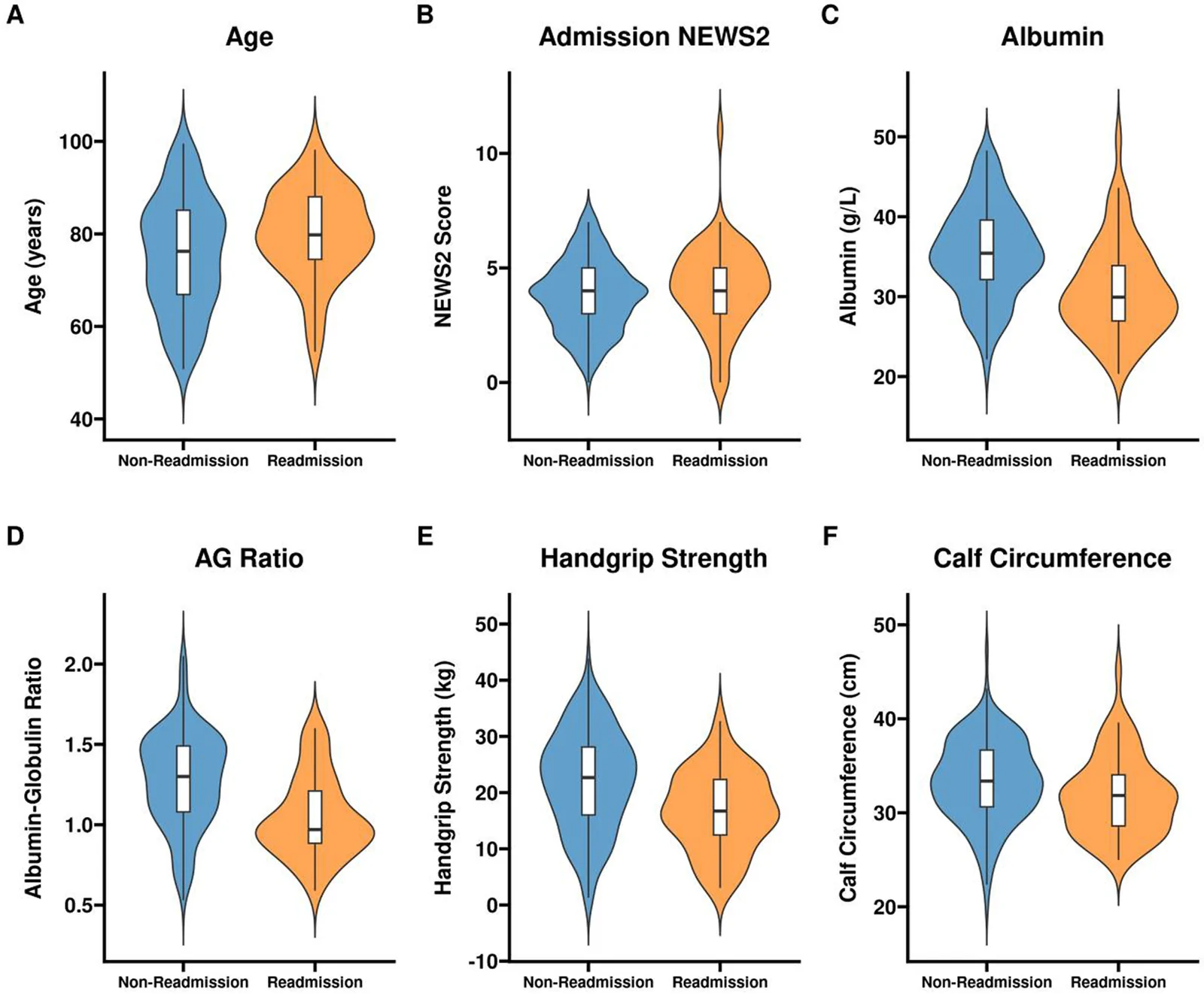
Comparison of baseline characteristics between readmission and non-readmission groups. **(A)** Age (years); **(B)** Admission NEWS2 Score; **(C)** Albumin (g/L); **(D)** Albumin-Globulin Ratio; **(E)** Handgrip Strength (kg); **(F)** Calf Circumference (cm). Blue indicates the Non-Readmission group, and orange indicates the Readmission group. The box plots inside the violin plots represent the median and interquartile ranges.

After excluding 15 in-hospital deaths from the primary readmission analysis, 244 patients were included (59 readmitted, 185 not readmitted). Compared with the patients without readmission, and the Charlson Comorbidity Index was higher, the difference was statistically significant (*P* < 0.05). In terms of laboratory indicators, the serum albumin level of the readmission group was lower, while the C-reactive protein level was higher, and the albumin/globulin ratio was lower (*P* < 0.05). In terms of function and muscle status, the Barthel Index, handgrip strength and calf circumference of the readmission group were significantly lower than those of the non-readmission group (*P* < 0.05). In addition, the proportion of patients with dysphagia, pressure injury and polypharmacy in the readmission group was also higher (*P* < 0.05). There was no significant difference in sex and distribution of primary diagnosis between the two groups (See Table 1 and Figure 2).

**Table 1:** Comparison of baseline characteristics between readmission and non-readmission groups.

Characteristic	Non-readmission (*n* = 185)	Readmission (*n* = 59)	Statistic	*P*-value
Age (years), Mean ± SD	74.32 ± 13.85	80.67 ± 11.42	*t* = 3.192	0.002
Female, *n* (%)	104 (56.2)	32 (54.2)	*χ*²=0.071	0.789
Charlson Comorbidity Index, M (IQR)	3 (2, 5)	5 (4, 7)	*Z* = 6.020	<0.001
Admission NEWS2 score, Mean ± SD	3.45 ± 1.78	4.67 ± 2.01	*t* = 4.440	<0.001
Primary Dx: Heart Failure, *n* (%)	47 (25.4)	19 (32.2)	*χ*²=1.047	0.306
Primary Dx: Infection, *n* (%)	61 (33.0)	21 (35.6)	*χ*²=0.137	0.711
Serum Albumin (g/L), Mean ± SD	35.12 ± 5.67	30.45 ± 6.23	*t* = 5.378	<0.001
C-reactive Protein (mg/L), M (IQR)	12.5 (5.8, 28.3)	38.6 (16.7, 75.4)	*Z* = 7.150	<0.001
Albumin/Globulin Ratio, Mean ± SD	1.28 ± 0.31	1.05 ± 0.29	*t* = 5.039	<0.001
Barthel Index (score), M (IQR)	75 (55, 90)	50 (30, 70)	*Z* = 5.915	<0.001
Body Mass Index (kg/m²), Mean ± SD	24.56 ± 4.21	23.12 ± 4.58	*t* = 2.239	0.026
Handgrip Strength (kg), Mean ± SD	22.34 ± 8.76	16.45 ± 7.89	*t* = 4.603	<0.001
Calf Circumference (cm), Mean ± SD	33.78 ± 4.12	31.23 ± 4.56	*t* = 4.032	<0.001
Dysphagia Present, *n* (%)	25 (13.5)	18 (30.5)	*χ*²=8.900	0.003
Pressure Injury Present, *n* (%)	8 (4.3)	8 (13.6)	*χ*²=6.226	0.013
Polypharmacy (≥5 meds), *n* (%)	67 (36.2)	34 (57.6)	*χ*²=8.453	0.003

### 3.2 Prevalence of probable sarcopenia in patients with different nutritional status and 90-day readmission rate in the combined group

The overall prevalence of Probable sarcopenia was 31.1%, and the prevalence increased significantly with the deterioration of nutritional status (from normal nutrition to malnutrition), and the difference was statistically significant (*P* < 0.001). There was a significant difference in the 90-day readmission rate between the groups (*P* < 0.001). The readmission rate in Group D was the highest (47.8%), while the readmission rate in Group A was the lowest (9.1%). See Table 2. Sensitivity analyses using alternative definitions of probable sarcopenia were performed. When probable sarcopenia was defined without requiring the SARC-F ≥ 4 criterion (i.e., low handgrip strength plus low calf circumference), the prevalence was 33.2%, and the adjusted odds ratio for Group D versus Group A remained significant (aOR 3.78, 95% CI: 1.65–8.66, *P* = 0.002). When AWGS 2019 cut-offs were applied to all three components, the prevalence was 29.5%, and the aOR for Group D was 4.12 (95% CI: 1.80–9.45, *P* < 0.001). These consistent findings support the robustness of the association between the combined phenotype and readmission.

**Table 2:** Prevalence of probable sarcopenia across nutritional status and 90-day readmission rates by combined group.

Grouping	Total *n* (%)	Probable sarcopenia Cases *n* (%)	*χ*² (between groups)	*P*-value	90-day readmission *n* (%)	*χ*² (between groups)	*P*-value
By Nutritional Status	244 (100)	76 (31.1)	15.101	0.001	59 (24.2)	–	–
Normal (MNA-SF 12–14)	64 (26.2)	9 (14.1)			8 (12.5)		
At Risk (MNA-SF 8–11)	101 (41.4)	32 (31.7)			21 (20.8)		
Malnourished (MNA-SF 0-7)	79 (32.4)	35 (44.3)			30 (38.0)		
By Combined Group	244 (100)	–	–	–	59 (24.2)	30.908	<0.001
Group A: Normal Nutrition, No Probable sarcopenia	55 (22.5)	0 (0)			5 (9.1%, 95% CI: 3.0–20.0)		
Group B: Abnormal Nutrition, No Probable sarcopenia	113 (46.3)	0 (0)			19 (16.8%, 95% CI: 10.4–25.0)		
Group C: Normal Nutrition, Probable sarcopenia	9 (3.7)	9 (100)			3 (33.3%, 95% CI: 7.5–70.1)		
Group D: Abnormal Nutrition, Probable sarcopenia	67 (27.5)	67 (100)			32 (47.8%, 95% CI: 35.4–60.3)		

In the sensitivity analysis using MNA-SF without the anthropometric item, the reclassification rate for the combined group assignment was 4.5% (11/244 patients). The adjusted odds ratio for Group D vs. Group A remained significant (aOR 3.67, 95% CI: 1.58–8.52, *P* = 0.003), confirming the robustness of the primary findings.

### 3.3 Comparison of baseline characteristics of patients with different combined nutrition-probable sarcopenia groups

**Figure 3:**
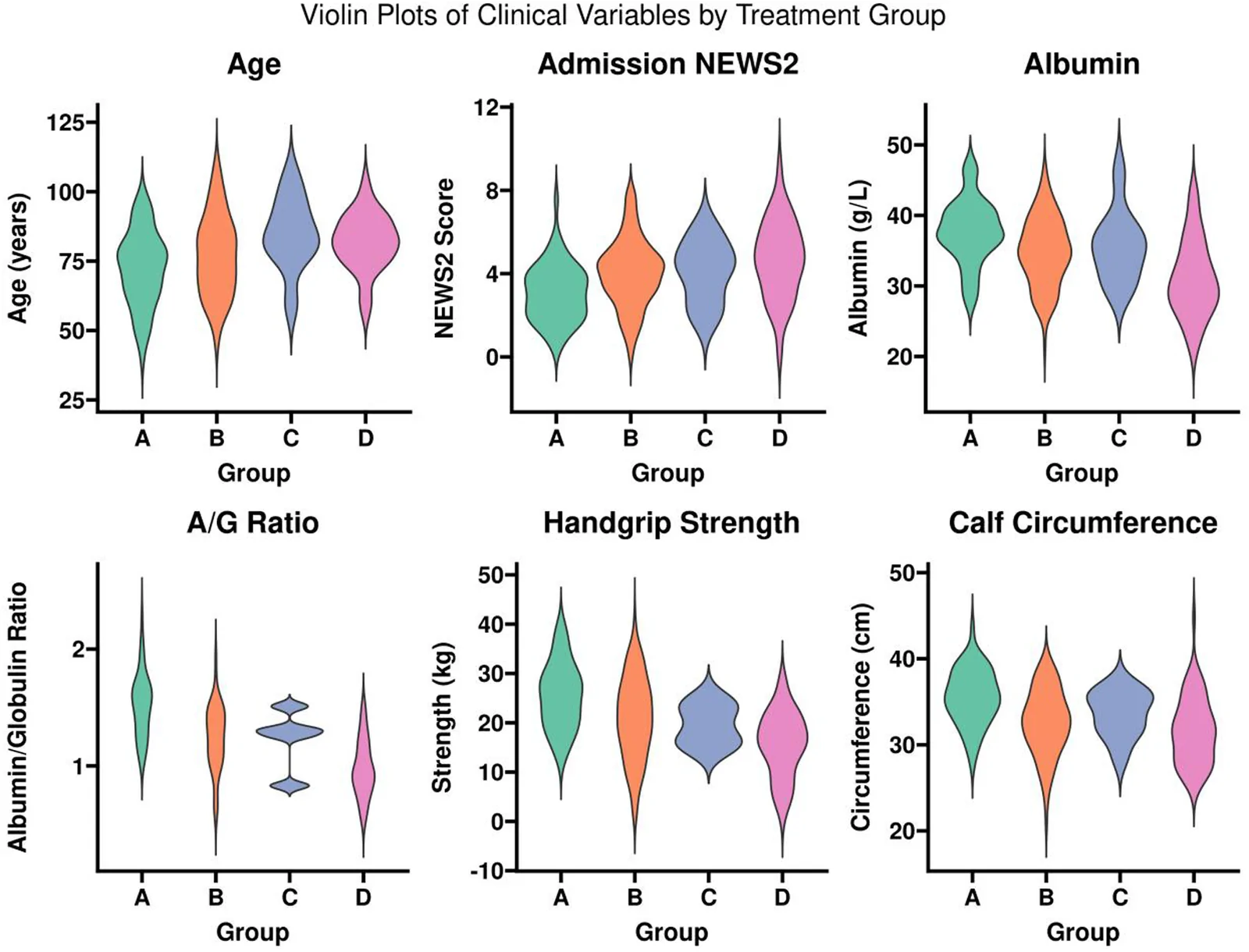
Comparison of baseline characteristics across different combined nutrition-probable sarcopenia groups.

There were significant differences in age, Charlson Comorbidity Index and C-reactive protein reflecting the level of inflammation among the groups (*P* < 0.05), and the values showed an increasing trend of deterioration from Group A to Group D. Similarly, there were significant differences in serum albumin, albumin/globulin ratio reflecting nutritional status, as well as Barthel Index, muscle strength (handgrip strength) and muscle mass (calf circumference) of functional status among groups (*P* < 0.05), and showed a decreasing trend from Group A to Group D. There was no difference in sex distribution among groups (See Table 3 and Figure 3).

**Table 3:** Comparison of baseline characteristics across different combined nutrition-probable sarcopenia groups.

Characteristic	Group A (*n* = 55)	Group B (*n* = 113)	Group C (*n* = 9)	Group D (*n* = 67)	Statistic	*P*-value
Age (years), Mean ± SD	70.23 ± 12.45	74.89 ± 13.21	79.33 ± 10.87	81.65 ± 11.56	*F* = 8.926	<0.001
Female, *n* (%)	31 (56.4)	65 (57.5)	5 (55.6)	35 (52.2)	*χ*²=0.487	0.921
CCI, M (IQR)	2 (1, 4)	3 (2, 5)	4 (3, 6)	5 (4, 7)	*H* = 36.452	<0.001
Admission NEWS2, Mean ± SD	3.01 ± 1.65	3.67 ± 1.82	3.89 ± 1.78	4.46 ± 2.10	*F* = 6.212	<0.001
CRP (mg/L), M (IQR)	8.2 (3.5, 18.0)	15.3 (7.0, 32.5)	14.8 (6.5, 30.0)	42.0 (20.5, 80.1)	*H* = 52.178	<0.001
Albumin (g/L), Mean ± SD	37.89 ± 4.23	34.56 ± 5.12	36.78 ± 4.89	30.01 ± 5.98	*F* = 24.780	<0.001
A/G Ratio, Mean ± SD	1.41 ± 0.28	1.25 ± 0.30	1.32 ± 0.27	1.02 ± 0.26	*F* = 20.040	<0.001
Barthel Index, M (IQR)	85 (70, 95)	70 (55, 85)	65 (50, 80)	45 (25, 65)	*H* = 87.231	<0.001
Handgrip Strength (kg), Mean ± SD	26.80 ± 7.45	21.73 ± 7.89	17.89 ± 6.23	15.12 ± 6.78	*F* = 25.911	<0.001
Calf Circumference (cm), Mean ± SD	35.51 ± 3.45	33.45 ± 3.78	32.11 ± 3.12	30.89 ± 4.23	*F* = 15.270	<0.001

### 3.4 Univariate and multivariate logistic regression analysis of factors affecting 90-day all-cause readmission

The logistic regression analysis results of influencing factors of 90-day all-cause readmission, the dependent variable was whether 90-day readmission occurred. Univariate analysis showed that age, higher Charlson Comorbidity Index, higher log-C-reactive protein level, lower serum albumin level, dysphagia and belonging to a specific combined group (especially Group D) were significantly associated with increased risk of readmission (*P* < 0.05). After adjustment by multivariate analysis, Charlson Comorbidity Index (*P* = 0.008) and log-C-reactive protein (*P* = 0.004) remained independent risk factors. In the combined group, only the group of “abnormal nutrition with Probable sarcopenia” (Group D) showed an independent and significantly increased risk of readmission (*P* = 0.001) (See Table 4). To test whether the effect of probable sarcopenia on readmission differed by nutritional status, a multiplicative interaction term (abnormal nutrition × probable sarcopenia) was introduced into the fully adjusted logistic model. The interaction was not statistically significant (coefficient = 0.468, standard erro*r* = 0.462, *P* = 0.312), indicating that the combined association does not depart significantly from multiplicativity. Formal additive interaction metrics (e.g., relative excess risk due to interaction) were not calculated because the small sample size limited reliable estimation.

**Table 4:** Univariate and multivariate logistic regression analysis of factors influencing 90-day all-cause readmission.

Variable	Univariate OR (95% CI)	*P*-value	Multivariate aOR (95% CI)	*P*-value
Age (per 1-year increase)	1.05 (1.02, 1.09)	0.003	1.03 (0.99, 1.07)	0.174
Female (vs. Male)	0.93 (0.52, 1.66)	0.804	0.88 (0.46, 1.69)	0.704
CCI (per 1-point increase)	1.30 (1.14, 1.48)	<0.001	1.22 (1.05, 1.41)	0.008
log-CRP (per 1-unit increase)	2.15 (1.51, 3.06)	<0.001	1.85 (1.22, 2.81)	0.004
Serum Albumin (per 1 g/L increase)	0.88 (0.83, 0.93)	<0.001	0.94 (0.88, 1.01)	0.093
Dysphagia Present (yes vs. no)	2.82 (1.43, 5.56)	0.003	1.72 (0.81, 3.67)	0.16
Combined Group (ref: Group A)		<0.001		0.008
Group B: Abnormal Nutrition, No Probable sarcopenia	2.02 (0.72, 5.68)	0.181	1.52 (0.68, 3.37)	0.304
Group C: Normal Nutrition, probable sarcopenia	4.95 (1.05, 23.36)	0.043	2.01 (0.41, 9.91)	0.384
Group D: Abnormal Nutrition, probable sarcopenia	9.24 (3.33, 25.64)	<0.001	3.95 (1.72, 9.08)	0.001

Given that Group C (normal nutrition with Probable sarcopenia) comprised only 9 patients with 3 readmission events, the estimates for this subgroup are imprecise (adjusted OR 2.01, 95% CI: 0.41–9.91) and should be interpreted descriptively. No firm conclusions regarding the independent effect of Probable sarcopenia in normally nourished patients can be drawn from these data. Model performance for the multivariate model: Hosmer–Lemeshow *χ*²=3.440, *P* = 0.904; bootstrap optimism-corrected AUC=0.698 (see Section 3.6 for details).

To assess the potential mediating effect of albumin on the nutrition-readmission association, we compared models with and without albumin adjustment. In the model without albumin, the aOR for Group D vs. Group A was 4.52 (95% CI: 2.01–10.17, *P* < 0.001). After adding albumin to the model, the aOR attenuated to 3.95 (95% CI: 1.72–9.08, *P* = 0.001), suggesting that albumin partially mediates but does not fully explain the association between combined malnutrition-Probable sarcopenia status and readmission. Albumin was retained in the fully adjusted model because it is also an acute-phase reactant reflecting systemic inflammation and illness severity, and its inclusion provides more conservative estimates of the nutritional effect.

NEWS2 was not included in the primary adjusted model because it is an acute severity-of-illness score measured at a single time point at admission and is highly correlated with both CRP (Spearman’s *ρ*=0.52, *P* < 0.001) and the combined nutrition-Probable sarcopenia groups (*P* < 0.001), raising concerns about collinearity and overadjustment. In a sensitivity analysis adding NEWS2 to the fully adjusted model, the aOR for Group D vs. Group A remained significant (aOR 3.21, 95% CI: 1.35–7.63, *P* = 0.008), and NEWS2 itself was not independently associated with readmission (aOR 1.15, 95% CI: 0.97–1.36, *P* = 0.112).

Similarly, the Barthel Index was excluded from the primary model because functional dependence lies on the causal pathway between sarcopenia/malnutrition and readmission, and its inclusion could represent overadjustment. In a sensitivity analysis adding the Barthel Index (as a continuous variable) to the fully adjusted model, the aOR for Group D vs. Group A was attenuated but remained significant (aOR 2.78, 95% CI: 1.15–6.72, *P* = 0.023), while the Barthel Index itself was associated with lower readmission odds (aOR per 10-point increase: 0.82, 95% CI: 0.70–0.96, *P* = 0.014).

### 3.5 Comparison of secondary clinical outcomes in patients with different combined groups

In-hospital mortality was low overall (15/244, 6.1%), limiting statistical power for between-group comparisons. Mortality was observed to be highest in Group D (9/67, 13.4%) and lowest in Group A (1/55, 1.8%); however, these findings are presented descriptively given the small number of events, and formal statistical inferences regarding mortality differences should be interpreted with caution. There was also a significant difference in the median length of hospital stay between groups (*P* < 0.05), showing a trend of gradual extension from Group A to Group D. The incidence of the 90-day composite endpoint (death or readmission) was significantly different among groups (*P* < 0.001), and the incidence in Group D was as high as 52.2% (See Table 5).

**Table 5:** Comparison of secondary clinical outcomes across different combined groups.

Outcome	Group A (*n* = 55)	Group B (*n* = 113)	Group C (*n* = 9)	Group D (*n* = 67)	Statistic	*P*-value
In-hospital Mortality, *n* (%)^a^	1 (1.8)	4 (3.5)	1 (11.1)	9 (13.4)	Fisher’s exact	0.029
Length of Stay (days), M (IQR)	7 (5, 10)	8 (6, 12)	9 (7, 14)	11 (8, 16)	*H* = 15.234	0.002
90-Day Composite Endpoint (Death or Readmission), *n* (%)^b^	6 (10.9)	21 (18.6)	4 (44.4)	35 (52.2)	Fisher’s exact	<0.001

a Denominator for in-hospital mortality is the full initial cohort (*N* = 259; Group A: 55, Group B: 113, Group C: 9, Group D: 67).

b Denominator for the 90-day composite endpoint is the full initial cohort (*N* = 259). The composite endpoint includes all-cause death (in-hospital or within 90 days post-discharge) and all-cause readmission within 90 days post-discharge.

### 3.6 Comparison of the effectiveness of different models in predicting 90-day readmission

**Figure 4:**
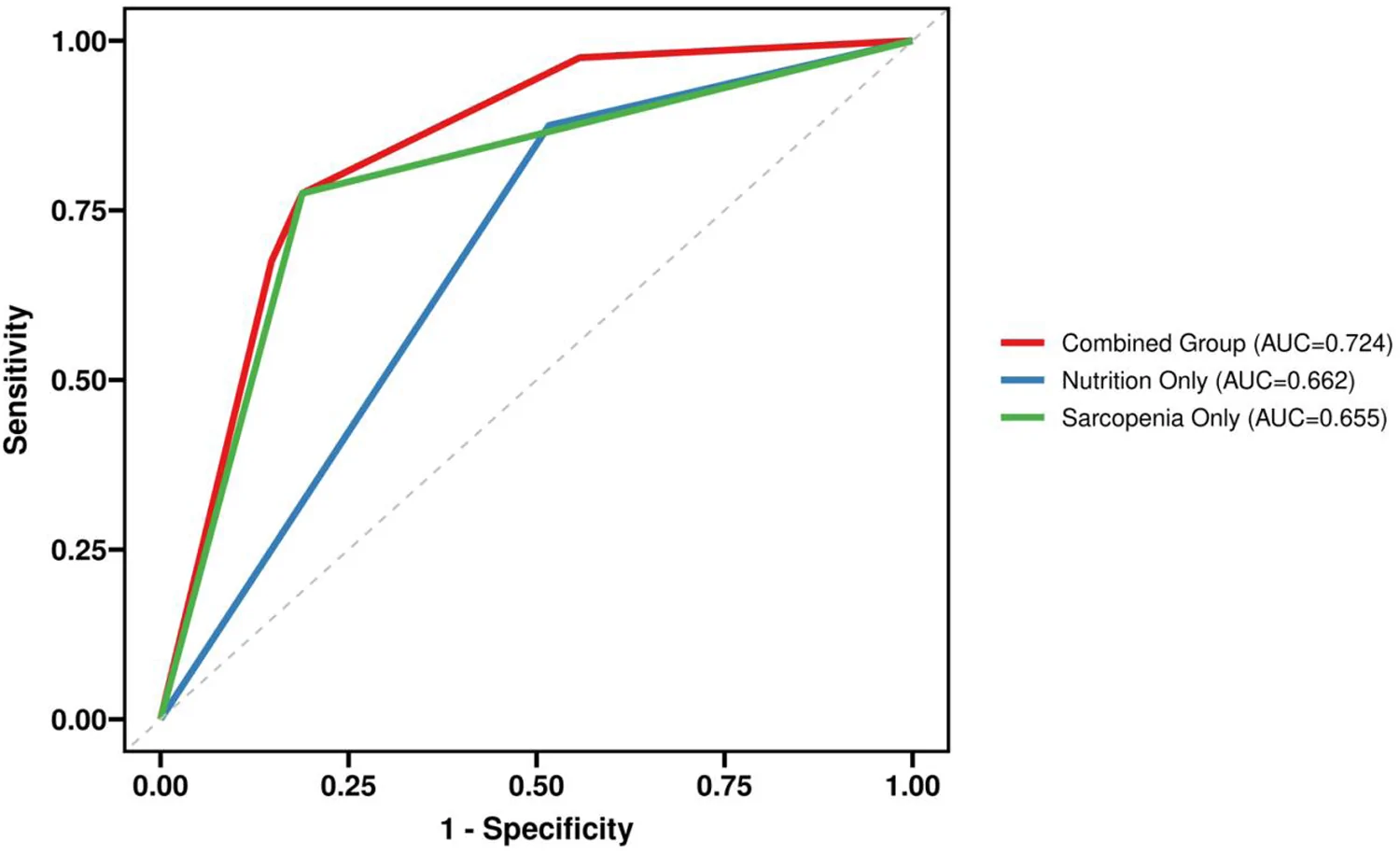
ROC of different models for 90-day readmission.

**Figure 5:**
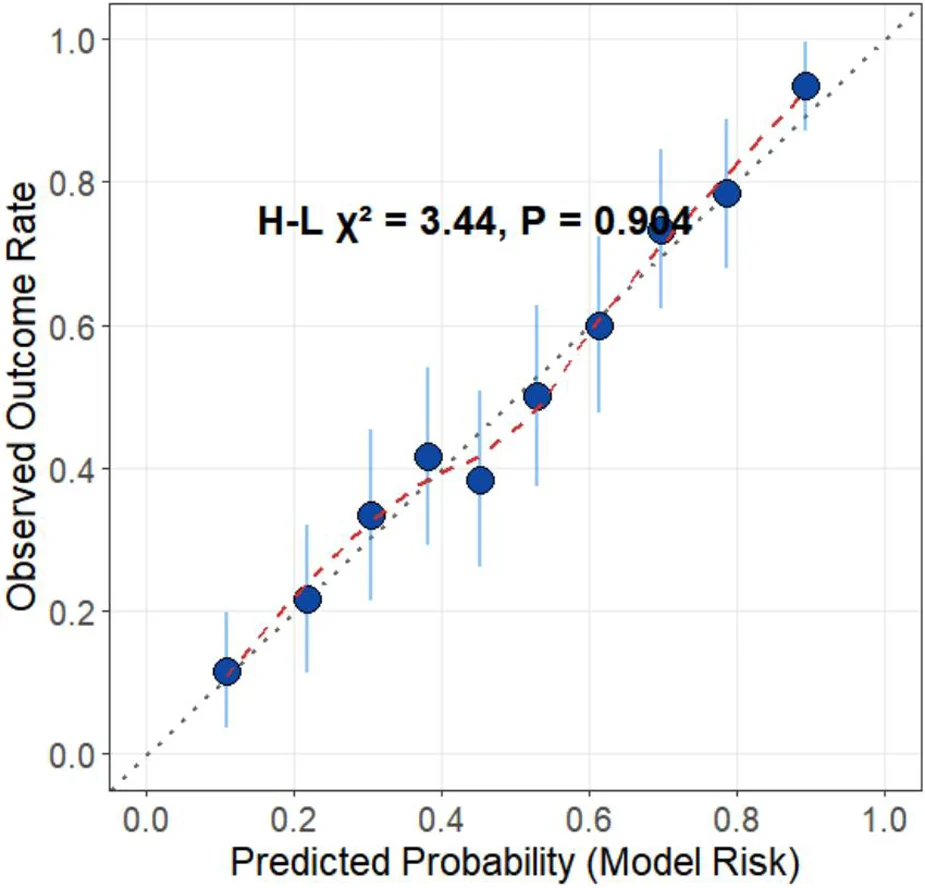
Calibration plot for the combined 4-category model.

The apparent AUC of the combined four-category model was 0.724 (95% CI: 0.651–0.797). Bootstrap internal validation with 1000 resamples yielded a mean optimism of 0.026, resulting in an optimism-corrected AUC of 0.698 (95% CI: 0.624–0.772) See Figure 4 and Table 6. The apparent calibration slope was 1.00 (by definition), and the optimism-corrected calibration slope was 0.91, indicating slight overfitting. The Hosmer–Lemeshow goodness-of-fit test for the combined model was not statistically significant (*χ*²=3.440, *P* = 0.904), indicating acceptable calibration, which confirms good agreement between predicted and observed risk across deciles of predicted probability. Pairwise model comparisons using the DeLong test were significant: Combined vs. Nutrition only, *Z* = 1.98, *P* = 0.048; Combined vs. probable sarcopenia only, *Z* = 2.06, *P* = 0.039. The calibration plot (Figure 5) visually confirms good agreement between predicted and observed risk, particularly in the lower and middle deciles of predicted probability. Due to the limited sample size and event rate, formal decision-curve analysis was not performed, as it may yield unstable estimates.

**Table 6:** Comparison of predictive performance of different models for 90-day readmission.

Prediction model	AUC (95% CI)	Sensitivity	Specificity	PPV	NPV
Combined Group (4-category)	0.724 (0.651–0.797)	54.20%	83.80%	47.80%	87.00%
Nutritional Status Only (MNA-SF 3-category)	0.662 (0.583–0.741)	50.80%	75.70%	34.40%	86.10%
Probable sarcopenia Diagnosis Only (Yes/No)	0.655 (0.576–0.734)	45.80%	82.20%	42.10%	84.40%

Pairwise comparisons with the combined four-category model were performed using the DeLong test. Combined vs. Nutrition only: *Z* = 1.98, *P* = 0.048; difference in AUC=0.062 (95% CI: 0.001–0.123). Combined vs. Probable sarcopenia only: *Z* = 2.06, *P* = 0.039; difference in AUC=0.069 (95% CI: 0.003–0.135).

## 4 Discussion

This study aims to explore the coexistence pattern of nutritional status and Probable sarcopenia, and its combined predictive value for the short-term readmission risk of elderly inpatients with multimorbidity. The main findings confirmed that the coexistence of abnormal nutritional status (MNA-SF ≤11) and Probable sarcopenia, rather than either condition alone, was independently associated with higher odds of readmission within 90 days after discharge. This combined state is closely related to heavier systemic inflammatory response, higher comorbidity burden and worse baseline functional reserve, and its predictive efficiency is better than that of evaluating nutritional or muscle status alone. The results emphasize the necessity of comprehensive evaluation of nutrition and function for identifying high-risk groups in elderly internal medicine patients (17).

Baseline characteristic analysis showed that patients with readmission exhibited the characteristics of older age, higher comorbidity index, more significant levels of inflammatory markers and worse physical function (18). This highly coincides with the connotation of the “frailty” phenotype in geriatrics, suggesting that readmission is not a single disease event, but a concentrated embodiment of individual vulnerability under the impact of acute disease. The coexistence of high levels of C-reactive protein and low albumin reflects the vicious cycle of chronic inflammation and nutrient depletion. This pathophysiological background may directly damage the ability of tissue repair and immune defense, increase the risk of acute complications, and drive the occurrence of readmission (19).

The high prevalence of malnutrition and Probable sarcopenia in this cohort, and the significant coexistence of both, highlights the prevalence of “malnutrition-inflammation-Probable sarcopenia” syndrome in elderly hospitalized patients (20). After combined grouping, nearly one third of patients were affected by both conditions simultaneously, and this group accounted for nearly half of the readmission events. This distribution pattern strongly suggests that there may be major omissions in clinical practice focusing only on either the nutrition or muscle dimension. Integrating both assessments can more accurately capture those patients who are extremely vulnerable due to the superposition of a variety of aging-related losses, which is of practical significance for risk stratification under conditions of limited resources (21).

The gradient of baseline characteristics across different combined groups clearly depicts a continuous spectrum of risk. Patients in Group D (coexisting abnormal nutrition and Probable sarcopenia) exhibited a phenotype of high vulnerability characterized by older age, greater comorbidity burden, stronger inflammatory activation, poorer nutritional indices, and worse functional status. Rather than representing an isolated effect of malnutrition and Probable sarcopenia, this group likely reflects an aggregation of multisystem impairments driven by the ‘malnutrition-inflammation-Probable sarcopenia’ syndrome, with each component both contributing to and resulting from the others. This high-vulnerability phenotype, rather than the individual components, is what drives the elevated readmission risk (22). This aggregation of multisystem damage is not accidental. Chronic disease-related inflammatory state can simultaneously lead to anorexia, hypermetabolism and increased protein breakdown, which jointly promote the process of malnutrition and muscle loss. Therefore, this state can be regarded as a serious “sickness syndrome”, and the underlying multisystem imbalance may be the root cause of the deterioration of clinical prognosis, with readmission being one of its external clinical endpoints (23).

In the multivariable analysis, the combined presence of abnormal nutritional status and probable sarcopenia was independently associated with higher odds of readmission. It is worth noting that abnormal nutrition without Probable sarcopenia (Group B) did not show a statistically significant independent association with readmission after full adjustment. The small size of Group C (normal nutrition with Probable sarcopenia, *n* = 9 with 3 events) precludes meaningful inference regarding this subgroup; the wide confidence interval (0.41–9.91) reflects this instability, and no conclusions should be drawn about the independent effect of Probable sarcopenia in normally nourished patients based on these data (24). The mechanism may be that both share the pathological basis of inflammation and metabolic disorders, and aggravate each other: malnutrition weakens the substrate supply for muscle protein synthesis, while the decreased activity and energy expenditure caused by Probable sarcopenia may further inhibit appetite and intake. In addition, the high comorbidity index and the level of inflammation remained independent risk factors, verifying the core role of multimorbidity and systemic inflammatory response in driving the reuse of healthcare resources (25).

The significant impact of combined grouping on in-hospital mortality, length of hospital stay, and composite endpoint further expands its clinical prognostic significance. Longer hospitalization time reflects the complexity of clinical conditions and delayed recovery, which may be related to difficulties in managing comorbidities, high incidence of complications, and slow functional recovery. The extremely high incidence of the composite endpoint indicates that this group not only faces risks during hospitalization, but also remains in an unstable state after discharge (26). This suggests that interventions for such patients need to go beyond acute phase treatment and include comprehensive management plans throughout hospitalization and after discharge, including nutritional support, rehabilitation training, and close follow-up, to break the vicious cycle of repeated hospitalizations (27).

The multiplicative interaction term between abnormal nutritional status and Probable sarcopenia was not statistically significant (*P* = 0.312 for the product term), suggesting that the combined effect does not depart significantly from multiplicativity. However, formal additive interaction metrics (such as the relative excess risk due to interaction, RERI) were not calculated. Therefore, the combined effect is best described as reflecting a joint association without specifying the precise nature of the interaction (additive or synergistic). The combined model yielded an AUC of 0.724, representing only moderate discriminative ability. The optimism-corrected AUC of 0.698 further highlights that the apparent performance may slightly overstate the true discriminative capacity. Thus, while the combined phenotype is a clinically meaningful risk marker, its standalone predictive accuracy is insufficient for definitive risk stratification; future models should incorporate additional prognostic variables (28). Future models may need to incorporate more dimensions, such as cognitive function, social support, or specific biomarkers, to construct more accurate predictive tools. In addition, the components of this model are simple and easily accessible, suggesting that, if externally validated, it could be considered for use in routine admission assessments to facilitate early identification of high-risk patients (29).

This study has a single-center retrospective design, and extrapolation of conclusions should be cautious. Although efforts have been made to control for key confounding factors, unmeasured confounding effects such as subtle differences in disease severity, socioeconomic factors, and specific treatment measures cannot be ruled out. Second, probable sarcopenia was defined using a modified three-criterion phenotype (SARC-F ≥ 4, low handgrip strength, and low calf circumference) without direct muscle mass measurement. Although handgrip strength and calf circumference are practical tools, they are surrogate measures and not as accurate as DXA or BIA. This phenotype corresponds to a high-specificity but low-sensitivity operationalisation of probable sarcopenia and cannot be equated with EWGSOP2-confirmed sarcopenia. The resulting misclassification may have attenuated the observed associations, and the true prognostic impact of Probable sarcopenia may be underestimated. Future multicenter, prospective studies are needed to validate the predictive efficacy of this combined grouping and explore comprehensive interventions targeting this specific high-risk group, such as combined nutritional support and resistance exercise, to determine whether they can effectively improve clinical outcomes and quality of life. Third, calf circumference was used as a surrogate for muscle mass rather than DXA or BIA as recommended by EWGSOP2. Edema, obesity, and acute illness may affect calf circumference measurements, potentially leading to misclassification. We attempted to minimize this by standardizing measurement conditions and recording edema status, but residual confounding from fluid status cannot be excluded. Fourth, the MNA-SF and our modified Probable sarcopenia criteria share overlapping constructs across several domains, including mobility, physical function, and anthropometric measurements (BMI and calf circumference). This conceptual and measurement overlap may artificially inflate the predictive performance of the combined model compared with either assessment alone, because the same or closely related patient characteristics contribute to both exposure classifications. Although our sensitivity analysis using MNA-SF with the anthropometric item removed yielded consistent results (aOR for Group D vs. Group A: 3.67, 95% CI: 1.58–8.52), this does not completely exclude the possibility of residual inflation, because the overlapping domains of mobility and physical function are also captured by the SARC-F questionnaire and handgrip strength assessment. Future studies using entirely non-overlapping assessment tools (e.g., GLIM criteria for malnutrition and imaging-based muscle mass measurement for sarcopenia) are needed to determine the true independent and joint effects of these two conditions. Until such studies are available, the prognostic value of the combined assessment reported here should be interpreted with this inherent limitation in mind. In addition to the text added for Comment 12): “Fourth, the MNA-SF, SARC-F, calf circumference, and Barthel Index share overlapping constructs related to mobility, physical function, and body composition. This conceptual and measurement overlap may partially inflate the predictive performance of the combined model compared with either assessment alone. Our sensitivity analysis using MNA-SF without the anthropometric item showed consistent results, suggesting that the overlap does not fully account for the observed associations. Fifth, the subgroup with normal nutritional status and probable sarcopenia (Group C) comprised only 9 patients with 3 readmission events, yielding an extremely imprecise adjusted odds ratio (95% CI: 0.41–9.91). This small cell size prevents any reliable inference about the independent impact of probable sarcopenia in normally nourished patients; all estimates for Group C should be interpreted descriptively. Larger, multicentre studies with more balanced exposure groups are required to clarify this relationship. Sixth, our Probable sarcopenia definition required simultaneous positivity on SARC-F, low handgrip strength, and low calf circumference, a combination that has not been validated against gold-standard muscle mass measurement. This modified phenotype likely differs from standard EWGSOP2-defined Probable sarcopenia, and its diagnostic performance characteristics (sensitivity, specificity) remain unknown. Consequently, the observed associations should be interpreted as pertaining to this specific high-specificity phenotype rather than to sarcopenia as defined by consensus criteria.”

In summary, in this single-center retrospective cohort of elderly multimorbid inpatients, the coexistence of abnormal nutritional status (MNA-SF ≤11) and Probable sarcopenia was independently associated with higher 90-day readmission. This high-vulnerability phenotype was characterized by greater comorbidity burden, heightened systemic inflammation, and poorer functional status. Larger prospective multicenter studies with direct muscle mass measurement, formal additive interaction testing, and assessment of targeted interventions are needed to validate this combined assessment strategy and determine its clinical utility for reducing readmission.

## Data Availability

The original contributions presented in the study are included in the article/Supplementary Material, further inquiries can be directed to the corresponding author.
